# Features of Structured, One-to-One Videoconference Interventions That Actively Engage People in the Management of Their Chronic Conditions: Scoping Review

**DOI:** 10.2196/58543

**Published:** 2025-02-26

**Authors:** Yu-Ting Chen, Michelle Lehman, Toni Van Denend, Jacqueline Kish, Yue Wu, Katharine Preissner, Matthew Plow, Tanya L Packer

**Affiliations:** 1 School of Occupational Therapy, Faculty of Health Dalhousie University Halifax, NS Canada; 2 Think Self-Management Inc. Halifax, NS Canada; 3 Department of Occupational Therapy University of Illinois Chicago Chicago, IL United States; 4 Center for Rehabilitation Outcomes Research Shirley Ryan AbilityLab Chicago, IL United States; 5 Department of Rehabilitation Medicine University of Minnesota-Twin Cities Minneapolis, MN United States; 6 Institute on Community Integration University of Minnesota-Twin Cities Minniapolis, MN United States; 7 Frances Payne Bolton School of Nursing Case Western Reserve University Cleveland, OH United States; 8 School of Health Administration Faculty of Health Dalhousie University Halifax, NS Canada; 9 Department of Nursing Umeå University Umeå Sweden

**Keywords:** videoconference, chronic disease management, active participation, intervention program, self-management, scoping review, Taxonomy of Every Day Self-Management Strategies, TEDSS, Behavior Change Technique Taxonomy version 1, BCTTv1, behavior change, mobile phone

## Abstract

**Background:**

A dramatic increase in the use of videoconferencing occurred as a response to the COVID-19 pandemic, including delivery of chronic disease management programs. With this increase, clients’ openness to and confidence in receiving any type of telehealth care has dramatically improved. However, the rapidity of the response was accomplished with little time to learn from existing knowledge and research.

**Objective:**

The purpose of this scoping review was to identify features, barriers, and facilitators of synchronous videoconference interventions that actively engage clients in the management of chronic conditions.

**Methods:**

Using scoping review methodology, MEDLINE, CINAHL, and 6 other databases were searched from 2003 onward. The included studies reported on structured, one-on-one, synchronous videoconferencing interventions that actively engaged adults in the management of their chronic conditions at home. Studies reporting assessment or routine care were excluded. Extracted text data were analyzed using thematic analysis and published taxonomies.

**Results:**

The 33 included articles reported on 25 distinct programs. Most programs targeted people with neurological conditions (10/25, 40%) or cancer (7/25, 28%). Analysis using the Taxonomy of Every Day Self-Management Strategies and the Behavior Change Technique Taxonomy version 1 identified common program content and behavior change strategies. However, distinct differences were evident based on whether program objectives were to improve physical activity or function (7/25, 28%) or mental health (7/25, 28%). Incorporating healthy behaviors was addressed in all programs designed to improve physical activity or function, whereas only 14% (1/7) of the programs targeting mental health covered content about healthy lifestyles. Managing emotional distress and social interaction were commonly discussed in programs with objectives of improving mental health (6/25, 24% and 4/25, 16%, respectively) but not in programs aiming at physical function (2/25, 8% and 0%, respectively). In total, 13 types of behavior change strategies were identified in the 25 programs. The top 3 types of strategies applied in programs intent on improving physical activity or function were feedback and monitoring, goals and planning, and social support, in contrast to shaping knowledge, regulation, and identity in programs with the goal of improving mental health. The findings suggest that chronic condition interventions continue to neglect evidence that exercise and strong relationships improve both physical and mental health. Videoconference interventions were seen as feasible and acceptable to clients. Challenges were mostly technology related: clients’ comfort, technology literacy, access to hardware and the internet, and technical breakdowns and issues. Only 15% (5/33) of the studies explicitly described compliance with health information or privacy protection regulations.

**Conclusions:**

Videoconferencing is a feasible and acceptable delivery format to engage clients in managing their conditions at home. Future program development could reduce siloed approaches by adding less used content and behavior change strategies. Addressing client privacy and technology issues should be priorities.

## Introduction

### Background

With the rise in popularity and availability of technology and the restrictions imposed by the COVID-19 pandemic, many health care providers have turned to telehealth care as an alternative, enhancement, or complement to face-to-face health care delivery [1]. Telehealth care refers to any remote communication or IT between clients and health care providers (eg, phone calls, SMS text messages, emails, and videoconferencing). In the United States, the use of telehealth care increased close to 30 times between September 2019 and September 2020, with many clients becoming first-time users during the pandemic [1]. As such, clients’ openness to and confidence in receiving any type of telehealth care has dramatically improved. For example, approximately 80% of clients report being satisfied with telehealth care, and 75% wish it to continue as a regular part of their care. However, the pandemic-driven shift to telehealth care was rapid and reactive to public health measures introduced to control the spread of the disease. For this reason, knowledge about effective practices, facilitators, and barriers to using telehealth care to support adults with chronic conditions is limited or incomplete. Without this knowledge, the integration of telehealth care into regular care will continue to be fragmented and reactive.

Synchronous videoconferencing has been proposed as “a viable alternative to face-to-face [health and medical] appointments” [2]. Using an internet-based platform, bidirectional audio and video signal is exchanged in real time (ie, synchronously) enabling verbal, nonverbal, and typewritten communication between health care providers and clients. A scoping review published in 2014 identified >500 studies using synchronous videoconferencing in client care between 2002 and 2012, with a steep upward increase in publications at the end of this period [3]. The review revealed that videoconferencing was used by a wide variety of health care professionals to make diagnoses, provide consultation, monitor client compliance or progress, and support clients in managing both acute and chronic physical and mental conditions. While this review provided a broad picture of how videoconferencing was being used, it did not distinguish between routine care visits and chronic condition management programs, nor did it investigate the content or active ingredients in the interactions or report the facilitators and barriers afforded by this format of service delivery. In-depth reviews in focused areas of clinical care are needed to inform optimal practice.

The Centers for Disease Control and Prevention reports that 60% of adults in the United States live with one or more chronic conditions, making chronic conditions the leading cause of death and disability and a key driver of annual health care costs [4]. Living with chronic conditions requires active engagement in ongoing medical, role, and emotional management [4,5]. This work, commonly termed self-management, includes seeking support, making decisions, and altering behaviors in the context of everyday life [6-8]. In contrast to passive receipt of educational information, active client participation is a hallmark of “informed, activated patients” described in the widely endorsed chronic care model [9,10]. The active involvement of individuals in these interventions sets them apart from many other medical interventions and warrants specific investigation.

### Objectives

Preventing chronic conditions by promoting and enabling self-management is now widely regarded as critical to improving client outcomes and reducing demands on health systems [6,7]. Therefore, in-person chronic disease management and self-management programs have proliferated. Many, particularly those for older adults, who are the most likely to have chronic conditions, are condition specific and have structured content that is delivered in a 6- to 8-week period, usually consisting of weekly sessions [11]. Self-management programs are known to effectively improve health outcomes and reduce health system demands [6,7]. As such, they have become a primary focus of health service policy, redesign, and research [9,12], with delivery both in person and via telehealth care. The purpose of this scoping review was to examine how videoconferencing has been used to deliver structured, synchronous, one-to-one interventions to clients in their own homes and actively engage them in the prevention and management of their chronic conditions.

## Methods

### Design

A scoping review is “a form of knowledge synthesis that addresses an exploratory research question aimed at mapping key concepts, types of evidence, and gaps in research related to a defined area or field” [13]. This methodology was selected specifically because the features of videoconferencing interventions to support adults with chronic conditions remain unclear. The 5-stage methodological framework by Arksey and O’Malley [14] was followed with augmentation consistent with recent evolutions of the scoping review methodology [15,16]. Focused on conceptual knowledge synthesis, scoping reviews do not typically assess the quality of the studies, nor are they intended to assess effectiveness. The PRISMA-ScR (Preferred Reporting Items for Systematic Reviews and Meta-Analyses extension for Scoping Reviews) checklist [17] proposed by the Enhancing the Quality and Transparency of Health Research Network was used to guide reporting of study conduct and findings (Multimedia Appendix 1). The protocol was developed and registered on the Open Science Framework [18]. Ethics approval was not sought or required for this review article.

### Identifying the Research Question

The research question was discussed and defined by an international research team comprising occupational therapists or researchers with expertise in chronic disease management. The agreed upon question—“What is known about the theoretical foundation, purpose and contents, active ingredients, program structure, technology, and facilitators and barriers of structured, synchronous, one-on-one videoconference interventions that actively engage clients who are living at home in the management of their chronic conditions?”—reflects the intent to help researchers and clinicians create, tailor, or transfer in-person interventions to internet-based delivery. The focus on structured, synchronous, one-on-one videoconferencing interventions was chosen because they most closely imitate in-person chronic disease interventions. Client location during the videoconference was restricted to their home.

Chronic conditions are defined broadly as “conditions that last one year or more and require ongoing medical attention or limit activities of daily living or both” [4]. In this review, we interpreted this to include populations medically at risk, such as people with obesity or frailty or requiring common interventions such as knee arthroplasty. Features such as program structure, asynchronous activities, videoconferencing technology, and program feasibility and acceptability were operationalized a priori (Textbox 1). An existing taxonomy and an existing framework were adopted to describe and categorize program content and behavior change strategies. The Taxonomy of Every Day Self-Management Strategies (TEDSS) [19] was used to operationalize program content. Active ingredients or strategies facilitating behavior change were operationalized using the Behavior Change Technique Taxonomy version 1 (BCT Taxonomy v1) hierarchical clusters [20] (see the Collating, Summarizing, and Reporting the Results section for details of each). Program purpose and theoretical foundation were not defined a priori, relying instead on the descriptions provided by each author. Similarly, barriers and facilitators were identified based on those reported in the text or quantitative reports of client satisfaction found in the study results.

**A priori operational definitions of key terms.**

**Term and operational definition or selected taxonomy**

- Chronic conditions: “conditions that last one year or more and require ongoing medical attention or limit activities of daily living or both” [4]
- Program structure: intervention duration, frequency, and asynchronous activities
- Asynchronous activities: activities undertaken outside or between the videoconferencing sessions, such as homework or symptom or activity monitoring
- Videoconferencing technology: software and hardware used or supplied, compatibility and connectivity requirements, data security, and any efforts made to set up or train clients to use the technology at home
- Program content: the Taxonomy of Every Day Self-Management Strategies framework [19]
- Active ingredients and behavior change strategies: Behavior Change Techniques taxonomy version 1 hierarchical clusters [20]
- Feasibility and acceptability: retention, attrition, and attendance rates

### Identifying Relevant Studies

The search strategy was developed and supported by a medical librarian with expertise in systematic and scoping reviews. Electronic databases in the field of health science and education (MEDLINE, PubMed, CINAHL, Embase, PsycINFO, OTseeker, PEDro, Cochrane, and ERIC) were searched using the following keywords—“videoconferencing,” “teleconferencing,” “virtual care,” “skype,” “teleconsultation,” “video call,” “telerehab,” “ehealth,” and “digital”—to identify videoconference interventions. These keywords reflect the wide array of terminology used to describe videoconferencing in health care interventions and client education. Because PsycINFO and ERIC often include healthy samples or populations, the keywords “patients,” “illness,” and “disease” were added to those searches. Keywords related to self-management were deliberately not included in the search strategies. Review of preliminary searches revealed that many interventions meeting the criteria of active engagement and self-management of chronic conditions were excluded when these keywords were added. Because their addition greatly reduced the number of studies found, selection based on active engagement was conducted during the title, abstract, and full-text screening processes. The search was limited to articles published in English. The initial search took place on June 3, 2020. In response to the proliferation of published literature, the search was repeated on January 25, 2023, adding the period from January 2020 to January 2023. The combined searches included articles reporting on adult samples published within the 20-year period between January 2003 and January 2023. Studies published before 2003 were excluded due to the limited currency of videoconferencing technology at that time. Detailed search strategies for each database and the number of hits from each are presented in Multimedia Appendix 2.

### Study Selection

Article inclusion and exclusion criteria were defined for (1) participants, (2) intervention program technology and format, and (3) intervention content and features (Table 1). Review studies and protocol papers were included. Review studies were further checked to identify missing articles fitting the selection criteria. Because protocol papers often provide rich information about program content, they were included to help map intervention structure, content, and active ingredients, recognizing that there would be no extractable data on facilitators and barriers. Routine care, defined as having regular meetings to diagnose or monitor clients’ conditions; compliance with a treatment or medication regime; or treatment without a predefined structure, topics, or modules during videoconferencing were excluded.

**Table 1:** Inclusion and exclusion criteria for the studies—videoconference interventions for people with chronic conditions.

Category and subcategory			Inclusion criteria		Exclusion criteria
**Participants**					
	Adults aged ≥18 years	Adults aged ≥18 years		Dyads of parents and children aged <18 years	
	Community dwelling	Community dwelling (eg, private home, supported housing, or group homes)		Individuals living in a hospital, long-term care facilities, or mental health facilities	
	One or more chronic conditions	Having at least one chronic or medically at-risk condition requiring ongoing medical attention for >1 year; the condition must impact activities of daily living (eg, heart condition, diabetes, obesity, or knee arthroplasty)		Caregivers as the focus of the intervention Health professionals or trainees as the focus of the intervention No indication of chronic conditions (eg, inactive adults, people seeking psychological consultations, smokers, and veterans)	
**Program technology and format**					
	One-on-one	Individual meeting in which the service provider saw 1 client or 1 client with their adult companion or caregiver		Group videoconference or one videoconference with multiple individuals at the same time (eg, educational session for multiple people)	
	Synchronous	Concurrent presence of the client or client and adult companion or caregiver and service provider in real time		Programs delivered completely asynchronously	
	Videoconferencing at home	Internet-based communication through transmitted audio and video signals; the client is located at home (for RCTs^a^, at least one arm had to meet this criterion)		Communication via telephone call only or combining telephone call and videoconferencing Videoconferencing outside the client’s home (eg, community center or clinics) Information transmission through virtual reality, robotic interventions, or platforms without interaction (eg, electronic monitoring system or automatic graphical feedback)	
**Intervention program content and features**					
	Structured program	Interventions with a stated goal or purpose and a structure (topics, modules, frequency, and duration of sessions)		Diagnostic and assessment-focused studies Routine care or follow-up sessions without structured intervention modules	
	Active client participation	Evidence of active client participation (eg, verbal, physical, or cognitive), including coaching and healthy behavior monitoring		Studies that only asked participants to listen to a lecture or read study materials (passive educational approach)	

^a^RCT: randomized controlled trial.

Study selection protocols for the initial search (June 2020) are described in detail. The protocols for the updated search (January 2023) were identical unless otherwise noted. At each time point, search results were imported into the Covidence platform (Veritas Health Innovation Ltd) for screening and selection. Covidence automatically removed duplicate articles. A total of 8 researchers participated in the review process (Multimedia Appendix 3). The lead reviewers (reviewers 3 and 6) together with reviewers 2, 4, and 5 were involved throughout both the initial and updated searches. Reviewers 1 and 2 were involved in the initial search. Reviewers 7 and 8 were involved in the updated search.

Before the initial title and abstract screening, interrater reliability trials were completed to ensure that reviewers were consistent in their selection. Reviewers 1 to 6 read the same 5 articles, independently deciding whether they met the inclusion and exclusion criteria. Conflicts were discussed, and operational definitions of the eligibility criteria were refined. This process was repeated until the κ interrater agreement level reached 0.8 [13,21]. With strong interrater reliability confirmed, title and abstract searches were completed by 1 of 5 reviewers (reviewers 2 to 6). Reviewers 3, 5, and 6 were joined by reviewers 7 and 8 to complete the updated title and abstract screening. New reviewers were provided with the written operational definitions and extensively oriented before commencing their reviews.

The full texts of retained studies were downloaded or ordered through interlibrary loan and imported into Covidence. All identified articles were located and retrieved. As with the initial title and abstract search, interrater reliability testing achieved a κ agreement of at least 0.8 before beginning the final selection of articles. In total, 2 reviewers independently screened each full-text article. For both the original and updated searches, conflicts were discussed and resolved by a full professor (reviewer 6) and a postdoctoral fellow (reviewer 3); for the updated search, conflicts were resolved by the same professor and an experienced research occupational therapist (reviewer 7).

A total of 94 studies met the inclusion criteria after the first full-text screening. Close examination revealed three distinct intervention types: (1) interventions specifically designed for individuals with mental health conditions (eg, depression, anxiety, and posttraumatic stress disorder), (2) solely exercise-based or repetitive learning interventions (eg, motor learning approach, muscle-strengthening exercises, and word-finding therapy) with no evidence of active client decision-making, and (3) interventions intended to support participants to actively and deliberately manage everyday life with a chronic condition. Exercise, activity-based, or repetitive interventions that explicitly included components to manage everyday life (eg, problem-solving, information seeking, and decision-making) were included in the third group. Given that existing reviews have reported on mental health populations [22-26] and exercise-based or repetitive interventions [27-29], this manuscript reports an analysis of the third group of interventions, for which there is less evidence.

During the original search, assignment of articles to the 3 intervention types was first completed by a single, consistent reviewer (reviewer 3); 4 other reviewers each assessed one-quarter of the articles, meaning that all articles were assigned by a consistent reviewer and one other. Conflicts were resolved by the lead reviewers. When the search was updated, reviewers 3 and 6 both assigned all articles to an intervention type and resolved conflicts through discussion.

### Charting the Data

Data extraction was completed in Covidence using extraction template 2.0. The extraction form was developed based on the research question and then pilot-tested by the lead reviewers, who independently extracted data from 2 of the included articles. The data extracted included general article information, research objectives, study design, participants (clients or recipients and clinicians or service providers), intervention purpose, content and features, videoconference technology and logistics, and facilitators of and barriers to delivering or receiving interventions using videoconference. A 2-hour workshop was held to familiarize team members with the data extraction process in Covidence. The 2 reviewers included for the updated search were similarly oriented. Double data extraction by 2 of 7 reviewers was then completed. The extracted data were checked, selected, or combined by 2 consistent reviewers for each search, resulting in comprehensive and rich data available for collation. Finally, the results were exported from Covidence to a CSV file and imported into Microsoft Excel for data analysis.

### Collating, Summarizing, and Reporting the Results

Extracted data were managed using Microsoft Excel. Categorical data (eg, study country, type of study design, and software and hardware selected) were analyzed using descriptive analysis. Text-based data were analyzed using either a known theoretical framework or thematic analysis. Intervention content and active ingredients were analyzed using the TEDSS framework [19] and the BCT Taxonomy v1 hierarchical clusters [20], respectively. The TEDSS framework, derived from the literature and interviews with 117 individuals living with chronic neurological conditions, was chosen for its robust and comprehensive categorization of strategies commonly used and considered important by people living with chronic conditions [19,30,31]. The 7 distinctive TEDSS domains (Textbox 2) have been used to successfully identify and describe content in self-management interventions [32,33], including in scoping reviews [11,34].

**Domain definitions—Taxonomy of Every Day Self-Management Strategies (TEDSS).**

**TEDSS domain and definition**

- Process strategies: strategies used to be well informed and make good decisions; often used to support use of other, nonprocess strategies
- Resource strategies: proactively seeking, pursuing, or managing needed formal or informal supports and resources
- Activity strategies: finding ways to participate in everyday activities (leisure activities, work activities, and household chores) despite problems such as fatigue, pain, memory loss, or disability
- Internal strategies: preventing and managing stress, negative emotions, and internal distress; creating inner calm
- Social interaction strategies: managing social interactions and relationships to be able to participate without exposure to negative reactions
- Health behavior strategies: maintaining a healthy lifestyle to enhance health and limit the risk of lifestyle-related illness
- Disease-controlling strategies: preventing, controlling, and limiting symptoms, complications, or disease progression

The BCT Taxonomy v1 [20] was used to categorize “observable, replicable, and irreducible component(s) of intervention[s] designed to alter or redirect causal processes that regulate behavior; that is, a technique proposed to be an ‘active ingredient’” [20]. The BCT Taxonomy v1 is a well-known taxonomy used to describe behavior change interventions. In this review, we used the 16-cluster definition developed by the BCT Taxonomy v1 authors to code the strategies used to facilitate clients’ behavior change [35].

All other text data (eg, theoretical foundation, intervention purpose, facilitators, and barriers) were coded and analyzed using thematic analysis. Keywords from the extracted text were first highlighted independently by 2 reviewers. Keywords were compared to generate themes before all extracted data were recoded using these themes. The extracted data (eg, intervention purpose, theoretical background, contents, and active ingredients) were cross-referenced to ensure that all the available data were included in the analysis. Data from both searches were combined before the number and percentage of programs assigned to each theme were calculated.

## Results

### Overview

**Figure 1:**
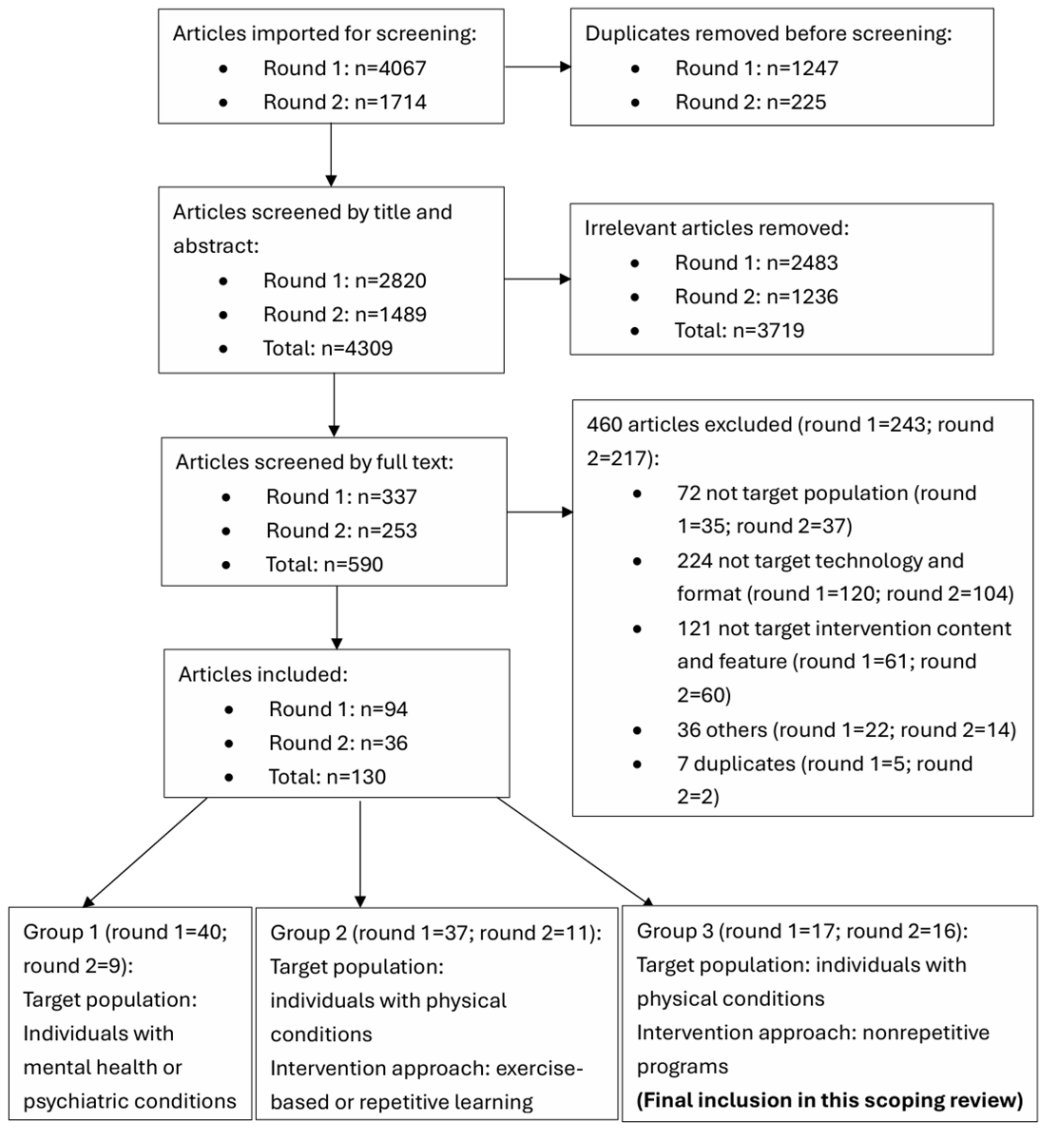
PRISMA (Preferred Reporting Items for Systematic Reviews and Meta-Analyses) flowchart.

The search of eligible articles from multiple databases generated 4067 potential articles in the initial search, with a further 1714 in the updated search. After removing duplicates, 4309 articles were screened by title and abstract, and 590 (13.69%) underwent full-text review. Of a total of 130 articles included after the full-text review, 33 (25.4%) [36-68] reported on self-management interventions for individuals with physical or cognitive impairments (ie, not solely exercise based or repetitive in nature or designed specifically for clients with a mental health diagnosis) and were included for final review (Figure 1).

A total of 48% (16/33) of the studies were conducted or designed in the United States (Table 2). In total, 18% (6/33) of the studies were conducted in Australia and Canada each, with the remaining conducted in Israel (3/33, 9%) and the United Kingdom and Ireland (1/33, 3% each). Over half of the papers reported randomized controlled trials (12/33, 36%) or were protocol papers (8/33, 24%), and a further 18% (6/33) were pilot or feasibility studies. Other designs were less well represented (Table 2). All the included studies (33/33, 100%) were published after 2012, with 85% (28/33) published after 2018. The 33 studies reported on 25 unique intervention programs (Table 3).

**Table 2:** Features of the studies included in the scoping review of videoconference interventions for people with chronic conditions (N=33).

Study	Year	Country	Study design	Participants				Service providers		Intervention
				Condition	Age (y)	Sample size, N^a^				
Alencar et al [36]	2019	United States	RCT^b^	Obesity	≥18	15	Multidisciplinary		TEAM^c^	
Alencar et al [37]	2020	United States	RCT	Obesity	≥18	13	Multidisciplinary		TEAM	
Aubin et al [38]	2019	Canada	RCT	Cancer	18-39	30	Psychologist		Psychosocial intervention	
Brunet et al [39]	2022	Canada	Protocol	Cancer	18-39	30-40	Physical activity counselor		ACCESS^d^	
Chemtob et al [40]	2019	Canada	RCT	Spinal cord injury	≥18	12	Kinesiologist		LTPA^e^	
Cruice et al [41]	2021	United Kingdom	Pilot or feasibility study	Stroke	37-81	29	Speech-language pathologist		Online SC^f^ for participation intervention	
Garland et al [42]	2021	Canada	Protocol	Cancer	≥18	124	Psychologist		CBT-I^g^	
Gilboa et al [43]	2019	Israel	Protocol	Hip fracture	≥60	30	Occupational therapist		CO-OP^h^	
Goren et al [44]	2022	Israel	RCT	IBD^i^	≥18	67	Social worker		COBMINDEX^j^ program	
Hastings et al [45]	2021	United States	RCT	Cognitive impairment or dementia	≥65	40	Nurse		Care management program	
Kelleher et al [46]	2019	United States	RCT	Cancer	≥18	89	Psychologist		PCST^k^	
Kline et al [47]	2019	United States	Protocol	Total knee arthroplasty	50-85	100	Multidisciplinary		Behavior change intervention	
Lafaro et al [48]	2020	United States	Quasi-experimental study	Cancer	≥65	34	Multidisciplinary		Telehealth perioperative physical activity intervention	
Lavelle et al [49]	2022	Ireland	Case report or case series	IBD	18-65	19	Psychologist		Brief and telehealth ACT^l^	
Lawson et al [50]	2020	Australia	Pilot or feasibility study	Stroke	≥18	28	Psychologist		Modified Monash memory skills program	
Lawson et al [51]	2022	Australia	Qualitative study	Stroke	≥18	34^m^	Psychologist		Modified Monash memory skills program	
Lynch et al [52]	2016	United States	Protocol	Diabetes	>45	30	Nurse		TABLETS^n^	
Milbury et al [53]	2020	United States	RCT	Cancer	≥18	75	Psychologist		CBM^o^	
Miller et al [54]	2017	United States	Protocol	Lower limb amputation	>50	32	Physiotherapist		PABC^p^	
Ng et al [55]	2013	Canada	Case report	TBI^q^	≥19	4	Occupational therapist		CO-OP	
Ownsworth et al [56]	2019	Australia	Protocol	Cancer	≥18	148	Psychologist		Tele-MAST^r^	
Pfammatter et al [57]	2022	United States	Protocol	Obesity	>18-70	—^s^	Health promotionist		EVO^t^	
Rietdijk et al [58]	2019	Australia	Pilot or feasibility study	TBI	≥18	2	Speech-language pathologist		TBIconneCT	
Rietdijk et al [59]	2020	Australia	RCT	TBI	18-70	19	Speech-language pathologist		TBIconneCT	
Somers et al [60]	2015	United States	Pilot or feasibility study	Cancer	≥19	25	Psychologist		PCST	
Tanenbaum et al [61]	2021	United States	Mixed methods	Diabetes	18-50	22	Psychologist		ONBOARD^u^	
Vellani et al [62]	2022	Canada	Pilot or feasibility study	Cognitive impairment or dementia	≥65	21	Nurse		VYV^v^	
Winger et al [63]	2020	United States	RCT	Cancer	≥19	89	Psychologist		PCST	
Winger et al [64]	2022	United States	Pilot or feasibility study	Cancer	≥18	30	Psychologist		MCPC^w^	
Wood et al [65]	2022	United States	Case report or case series	Spinal cord injury	≥18	15	Dietitian		Tele–nutrition counseling program	
Ymer et al [66]	2022	Australia	RCT	TBI or stroke	23-71	50^x^	Psychologist		CBT-SF^y^	
Beit Yosef et al [67]	2022	Israel	RCT	TBI	≥18	8	Occupational therapist		Tele–CO-OP	
Yuen [68]	2013	United States	Quasi-experimental study	Spinal cord injury	≥19	16	Occupational therapist		Telecare on oral health	

^a^For protocol papers, the target sample size is reported.

^b^RCT: randomized controlled trial.

^c^TEAM: Telehealth-Enabled Approach to Multidisciplinary Care.

^d^ACCESS: Physical Activity Counseling for Young Adult Cancer Survivors.

^e^LTPA: Leisure Time Physical Activity.

^f^SC: supported conversation.

^g^CBT-I: cognitive behavioral therapy for insomnia.

^h^CO-OP: Cognitive Orientation to Daily Occupational Performance.

^i^IBD: inflammatory bowel disease.

^j^COBMINDEX: Cognitive Behavioral and Mindfulness-Based Stress Reduction With Daily Exercise.

^k^PCST: Pain Coping Skill Training.

^l^ACT: acceptance and commitment therapy.

^m^25 stroke survivors and 9 clinicians.

^n^TABLETS: Tablet-Aided Behavioral Intervention Effect on Self-Management Skills.

^o^CBM: couple-based meditation.

^p^PABC: Physical Activity Behavior Change.

^q^TBI: traumatic brain injury.

^r^Tele-MAST: Making Sense of Brain Tumour program.

^s^Not available.

^t^EVO: Elements Vital to Treat Obesity.

^u^ONBOARD: Overcoming Barriers and Obstacles to Adopting Diabetes Devices.

^v^VYV: Voice Your Values.

^w^MCPC: Meaning-Centered Pain Coping Skill Training.

^x^50 participants began the program, and 30 completed it.

^y^CBT-SF: cognitive behavioral therapy for sleep disturbance and fatigue.

**Table 3:** Structure, foundation, and objectives of structured videoconference intervention programs.

Intervention name	Total number of sessions	Frequency	Duration of session	Intervention objective	Theoretical foundation	Asynchronous component
Behavior change intervention [47]	≥10	Weekly	≤1 hour	Improve physical activity or physical function	Social cognitive theory or CBT^a^ Control theory	Self-monitoring and tracking
PABC^b^ [54]	≥10	Weekly	≤1 hour	Improve physical activity or physical function	Behavior change	Self-monitoring and tracking
LTPA^c^ [40]	5-9	Weekly	≤1 hour	Improve physical activity or physical function	Self-determination theory	Not reported
TABLETS^d^ [52]	5-9	Weekly+booster sessions	≤1 hour	Improve physical activity or physical function Improve quality of life Other (diet, medication adherence, and self-monitoring behavior)	Previously reported intervention	Educational materials or access to website Printable materials or manuals Self-monitoring and tracking Automated feedback
ACCESS^e^ [39]	5-9	Not reported	≤1 hour	Improve physical activity or physical function	Behavior change Self-determination theory	Educational materials or access to website Home practice or homework
Telehealth perioperative physical activity intervention [48]	≤4	Other than weekly	Not reported	Improve physical activity or physical function	Chronic care self-management model	Printable materials or manuals Self-monitoring and tracking Other
Psychosocial intervention [38]	≤4	Other than weekly	≤1 hour	Improve mental health or reduce stress Improve quality of life	Review of literature or clinical guidelines	Printable materials or manuals
Tele-MAST^f^ [56]	≥10	Weekly	≤1 hour	Improve mental health or reduce stress Improve quality of life	Review of literature or clinical guidelines Sense of coherence theory	Printable materials or manuals
COBMINDEX^g^ program [44]	5-9	Other than weekly	>1 hour	Improve mental health or reduce stress	Social cognitive theory or CBT	Educational materials or access to website Home practice or homework Self-monitoring and tracking Other
Brief and telehealth ACT^h^ [49]	≤4	Weekly	>1 hour	Improve mental health or reduce stress	ACT	Printable materials or manuals
CBM^i^ [53]	≤4	Weekly	≤1 hour	Improve mental health or reduce stress	Previously reported intervention	Printable materials or manuals Home practice or homework Other
TEAM^j^ [36,37]	≥10	Weekly	≤1 hour	Weight loss	Not reported	Self-monitoring and tracking
Tele–nutrition counseling program [65]	5-9	Other than weekly	≤1 hour	Weight loss Improve quality of life	Health belief model	Educational materials or access to website Home practice or homework Self-monitoring and tracking
EVO^k^ [57]	≥10	Other than weekly	≤1 hour	Weight loss Diabetes management	Behavior change Psychoeducation	Educational materials or access to website Home practice or homework Self-monitoring and tracking
TBIconneCT [58,59]	≥10	Weekly	>1 hour	Improve communication	Previously reported intervention Social cognitive theory or CBT	Printable materials or manuals Home practice or homework
Online SC^l^ for participation [41]	≥10	Other than weekly	≤1 hour	Improve communication Improve quality of life Improve mental health or reduce stress Improve everyday participation or life roles	Previously reported intervention	Printable materials or manuals Message board or social networking site
PCST^m^ [46,60,63] and and MCPC ^c^ [64]	≤4	Not reported	≤1 hour	Pain management	Social cognitive theory or CBT	Educational materials or access to website Home practice or homework Self-monitoring and tracking Message board or social networking site
CO-OP^o^ [43,55,67]	≥10	Other than weekly OR weekly^p^	≤1 hour	Improve everyday participation or life roles	Meichenbaum’s SIT^q^ or CO-OP	Home practice or homework
Modified Monash memory skills program [50,51]	5-9	Weekly+booster sessions	>1 hour	Improve everyday participation or life roles Improve memory	Previously reported intervention	Home practice or homework
Telecare on oral health [68]	5-9	Other than weekly	≤1 hour	Improve oral care	Not reported	Other
Care management program [45]	≥10	Other than weekly	Not reported	Care management and planning Improve physical activity or physical function	Review of literature or clinical guidelines	Not reported
VYV^r^ [62]	≤4	Not reported	Not reported	Care management and planning	Behavior change Review of literature or clinical guidelines Representational approach to patient education	Other
ONBOARD^s^ [61]	≤4	Other than weekly	Not reported	Diabetes management	Review of literature or clinical guidelines Technology acceptance model	Not reported
CBT-I^t^ [42]	5-9	Weekly	≤1 hour	Improve sleep	Social cognitive theory or CBT	Home practice or homework Self-monitoring and tracking
CBT-SF^u^ [66]	5-9	Weekly	≤1 hour	Improve sleep Improve mental health or reduce stress	Social cognitive theory or CBT	Not reported

^a^CBT: cognitive behavioral therapy.

^b^PABC: Physical Activity Behavior Change.

^c^LTPA: Leisure Time Physical Activity.

^d^TABLETS: Tablet-Aided Behavioral Intervention Effect on Self-Management Skills.

^e^ACCESS: Physical Activity Counseling for Young Adult Cancer Survivors.

^f^Tele-MAST: Making Sense of Brain Tumour program.

^g^COBMINDEX: Cognitive Behavioral and Mindfulness-Based Stress Reduction With Daily Exercise.

^h^ACT: acceptance and commitment therapy.

^i^CBM: couple-based meditation.

^j^TEAM: Telehealth-Enabled Approach to Multidisciplinary Care.

^k^EVO: Elements Vital to Treat Obesity.

^l^SC: supported conversation.

^m^PCST: Pain Coping Skill Training.

^n^MCPC: Meaning-Centered Pain Coping Skills Training.

^o^CO-OP: Cognitive Orientation to Daily Occupational Performance.

^p^A total of 12% (4/33) of the studies tested the same intervention and delivered it on both a weekly and nonweekly schedule.

^q^SIT: self-instructional training.

^r^VYV: Voice Your Values.

^s^ONBOARD: Overcoming Barriers and Obstacles to Adopting Diabetes Devices.

^t^CBT-I: CBT for insomnia.

^u^CBT-SF: CBT for sleep disturbance and fatigue.

### Technology

Technology differed across the studies, even those reporting the same intervention program. The most common device used for videoconferencing was an iPad or tablet (12/33, 36%). Computers (6/33, 18%), any available device (3/33, 9%), and smartphones (3/33, 9%) were less common. Skype (9/33, 27%) and Zoom (9/33, 27%) were the most frequently used commercial platforms, with a variety of other commercial and custom-designed platforms reported (Multimedia Appendix 4 [36-68]).

Device source and ownership varied. Researchers provided all devices in some studies (9/33, 27%), whereas participants used their own devices in others (5/33, 15%). Finally, in some studies (4/33, 12%), researchers provided devices only if required. A total of 36% (12/33) of the studies did not report device type, and 45% (15/33) did not provide information on device ownership. Interestingly, 55% (18/33) of the studies stated that lack of access to a device, the internet, or a videoconference platform was a reason for exclusion. In total, 45% (15/33) included only participants who owned or had access to compatible devices and a reliable internet connection, excluding those without. A total of 24% (8/33) of the studies excluded participants who were unable to set up or log into the videoconference system independently or with assistance from family or friends.

In total, 15% (5/33) of the studies reported that the platform or software used was compliant with national or provincial personal and health information regulations, such as Health Insurance Portability and Accountability Act (HIPAA) or the Personal Information Protection and Electronic Documents Act (PIPEDA). A total of 27% (9/33) of the studies [36,37,39,41,45,55,62,64,68] reported strategies to enhance data security (eg, password-protected log-in procedures and encrypted data access), and 9% (3/33) [58,60,62] reported how participants were informed of data security and consented to any potential risk.

In total, 39% (13/33) of the studies confirmed participant device videoconference compatibility and internet strength. Training sessions (10/33, 30%) [37,40,41,45,48,52,55,56,65,68], securing family supports (4/33, 12%) [36,43,55,56], providing manuals (5/33, 15%) [38,45,55,58,63], home visits (2/33, 6%) [41,43], and email instructions (3/33, 9%) [45,58,68] were reported as ways to prepare and educate participants.

### Intervention Programs

A total of 33 studies met the inclusion criteria and reported on 25 unique intervention programs. Therefore, program data were aggregated and are presented by program rather than by study in this section.

Programs were delivered by various health care professionals, with psychologists (9/25, 36%) being the most common, followed by multidisciplinary health care teams and nurses (3/25, 12% each) and speech-language pathologists and occupational therapists (2/25, 8% each; Table 2). Participants were people with neurological conditions (10/25, 40%); cancer (7/25, 28%); musculoskeletal conditions (3/25, 12%); or inflammatory bowel disease, obesity, and diabetes (2/25, 8% each). One intervention was delivered to participants with both neurological *and* musculoskeletal conditions. A total of 32% (8/25) of the programs required a companion to join the videoconference to practice communication skills, facilitate family support and relationships, or promote activity engagement [43,45,48,53,55,56,58,59,62,65].

The number of sessions was almost evenly divided between 3 categories. In total, 36% (9/25) of the programs were delivered in ≥10 sessions, and another 36% (9/25) spanned 5 to 9 sessions. A total of 28% (7/25) of the programs had ≤4 sessions (Table 3). Weekly meetings for ≤1 hour were the most common arrangement (9/25, 36%). Only 16% (4/25) of the programs had videoconferencing sessions longer than an hour. A total of 24% (8/33) of the studies reported participant attendance rates of 71% to 100% of sessions, with 12% (4/33) reporting 100% attendance rates. Reported attrition rates ranged between 0% and 35% (10/33, 30% of the studies; protocol papers excluded).

Almost all programs (21/25, 84%) included numerous asynchronous elements in addition to the videoconferencing sessions (Table 3). Active learning elements such as self-monitoring and tracking or between-session practice were commonly reported (10/25, 40% of the programs each), as were more passive learning elements such as printed materials or manuals and access to websites (14/25, 56% of the programs).

On the basis of explicitly stated program goals or objectives, thematic analysis identified 12 distinct program objectives across the 25 intervention programs, with 9 (36%) programs indicating more than one objective (Table 3). Even though programs designed specifically for people with mental health diagnoses and solely exercise-based programs were excluded, the 2 most common objectives were improving physical activity or function and improving mental health, each reported by 28% (7/25) of the programs. The next most frequently reported objective was improving quality of life (5/25, 20%); however, this was rarely the sole program objective. Program-specific health objectives were also found, such as weight loss or improving oral health.

No single theoretical foundation dominated program development. Thematic analysis revealed that the social cognitive theory or cognitive behavioral therapy was the most cited theoretical foundation (6/25, 24%), followed by unspecified behavior change theories (4/25, 16%; Table 3). Studies also cited reviews of the literature or clinical guidelines (5/25, 20%) or previous interventions (5/25, 20%) as program foundations.

**Figure 2:**
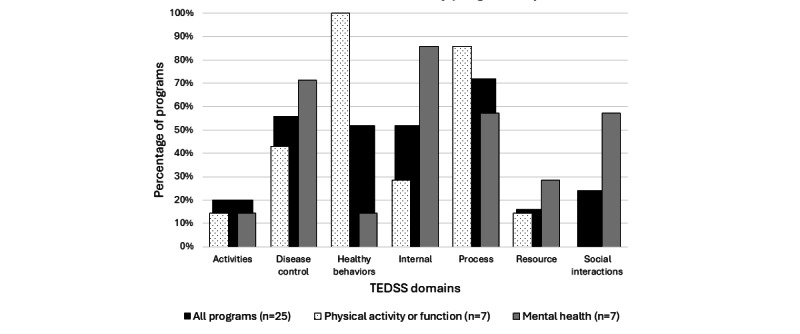
Intervention program content by program objective. TEDSS: Taxonomy of Every Day Self-Management Strategies.

No program included content from all TEDSS domains (Multimedia Appendix 5). However, the use of some domains was more common (Figure 2). In total, ≥50% of the programs (18/25, 72%) included content from the *process* domain (eg, finding information, problem-solving, decision-making, and action planning), the *disease control* domain (14/25, 56%; eg, medication and symptom management), the *healthy behaviors* domain (13/25, 52%; eg, diet and exercise), and the *internal* domain (13/25, 52%; eg, staying positive and reducing stress). In contrast, <25% of the programs included content from the other domains (Figure 2).

The diversity of the programs prevented a descriptive analysis based on client group or theoretical foundation; however, descriptive analysis based on 2 program objectives (improving mental health and improving physical activity or function) was possible and demonstrated unique patterns in the included content. Figure 2 illustrates markedly different content in 3 specific TEDSS domains. In total, 100% (7/7) of the interventions with the goal of improving physical activity or function included content on diet, exercise, or sleep hygiene (*healthy behavior* domain) compared to only 14% (1/7) of the interventions intended to improve mental health. The opposite trend was observed for the *internal* and *social interaction* domains—interventions with the goal of improving mental health frequently included content from these domains, whereas those focused on improving physical function did not. In fact, no programs with the goal of improving physical activity or function included content from the *social interaction* domain.

**Figure 3:**
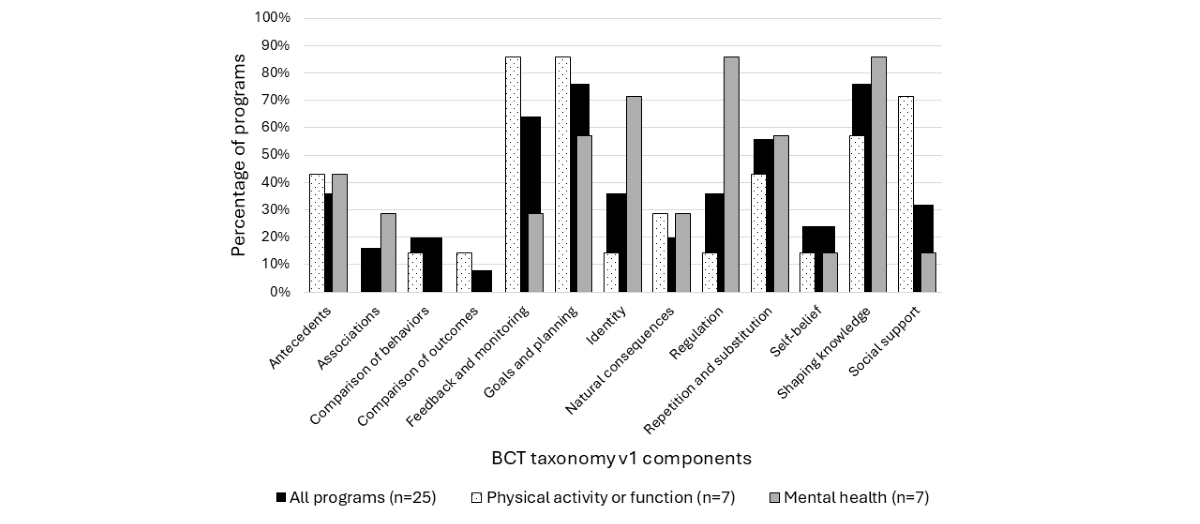
Behavior change components by program objective. BCT taxonomy v1: Behavior Change Technique Taxonomy version 1.

Of the 16 BCT Taxonomy v1 strategy groups, 4 (25%; goals and planning, shaping knowledge, feedback and monitoring, and repetition and substitution) were included in >50% of the intervention programs (goals and planning: 19/25, 76%; shaping knowledge: 19/25, 76%; feedback and monitoring: 16/25, 64%; repetition and substitution: 14/25, 56%), and 3 (19%) were not included at all (Figure 3). None of the programs reported using reward and threat, scheduled consequences, or covert learning techniques. As with program content, analysis based on program objective yielded different patterns of behavior change elements. In programs intent on improving physical activity or function, the feedback and monitoring, goals and planning, and social support strategies dominated. However, in programs with the goal of improving mental health, the shaping knowledge, regulation, and identity behavior change strategies were the most common.

### Facilitators of and Barriers to Videoconference Interventions

Text and quantitative results describing facilitators and barriers to using videoconferencing were extracted from 76% (25/33) of the articles (protocol papers were excluded). Health care provider perspectives were reported in only 6% (2/33) of the studies; therefore, only participant perspectives are reported. Five facilitators were categorized and labeled as follows:

1. Feasibility and acceptability to participants (11/25, 44%)
2. Reduced temporal and physical barriers (11/25, 44%)
3. Therapeutic benefit or therapeutic alliance (10/25, 40%)
4. Improved reach to underserviced populations or areas (6/25, 24%)
5. Allowing COVID-19 safety precautions (1/25, 4%)

Although 30% (10/33) of the papers were published in either 2021 or 2022, only 3% (1/33) of the papers [44] identified videoconferencing as a specific benefit during the period of COVID-19 restrictions. Definitions and sample quotes for each facilitator are presented in Table 4. Barriers to delivering interventions using videoconferencing were also identified and are described in Table 4:

1. Reliance on client comfort and technology literacy (8/25, 32%)
2. Limited access to technology and the internet (7/25, 28%)
3. Technical issues or breakdowns (7/25, 28%)
4. Protection of privacy and confidentiality (5/25, 20%)
5. Limited clinical observations and communication (3/25, 12%)

**Table 4:** Facilitators and barriers of videoconferencing interventions for people with chronic conditions (N=25).

Facilitators or barriers		Interventions, n (%)	Description	Examples
**Facilitators**				
	Feasible and acceptable to participants [37,38,45,46,48,51,53,58,60,63,65]	11 (44)	Low attrition rate and high completion rate Positive client feedback—easy to follow, practical, and convenient; easy and quick to reschedule if needed	“The delivery of the TBIconneCT intervention via videoconferencing was feasible and well received by the participants.” [58]
	Reduced temporal and physical barriers [37,38,40,44,46,51,60,61,63-65]	11 (44)	Saved time, cost, and energy required traveling to the clinic Avoidance of barriers in transportation, parking, and accessibility Particularly beneficial for individuals with greater difficulties traveling or with poorer health conditions	“The ease of completing sessions at home may have reduced treatment access barriers often reported by patients with cancer, such as difficulty with travel and parking.” [63]
	Therapeutic benefits from receiving the intervention at home or therapeutic alliance building [36,37,40,45,51,58,60,61,65,68]	10 (40)	Ability to observe and provide environmental recommendations at clients’ homes Direct application and practice of skills in naturalistic settings; no need to generalize skills learned in a clinical or laboratory setting Meaningful collaborative partnerships	“The first benefit was patient engagement and communication. Video was novel and fun for some patients, more interactive than telephone, and nurses felt they got to know video patients better...Study nurses reported the video was useful for some indications, such as viewing a drawer of medications or observing a patient doing shoulder exercises.” [45]
	Improved reach to underserviced populations or underserviced areas [38,51,60,62,63,65]	6 (24)	Improvement of health care service delivery in remote areas for clients with poor health	“Participants connected from diverse locations, including rural and urban areas, spread over large geographic distances. This widespread reach would not have been possible had the intervention taken place in-person.” [62]
	COVID-19 [44]	1 (4)	Allowing COVID-19 safety precautions	“In the current COVID-19 pandemic, where social distancing is an essential policy in many countries to decrease the spread of the disease, online interventions have become increasingly common.” [44]
**Barriers**				
	Reliance on patients’ comfort and technology literacy [38,40,41,45,46,58,63,68].	8 (32)	Limited confidence, comfort, and skills using technology Reduced trustworthiness of quality health care services	“In our sample of participants with CI, not unexpectedly, we found that adopting more than one new technology at a time was especially difficult for participants (e.g., learning how to use both the iPad and the video visit software).” [45]
	Limited access to compatible technology and the internet [36,40,41,46,55,62,68]	7 (28)	Home device or software is not compatible with the requirements of videoconferencing Insufficient internet bandwidth and upload or download speed High cost of better devices and signal quality	“Participants needed to be familiar with using technology and have access to a computer, limiting the accessibility of this design to all members of the SCI community.” [40]
	Technical issues [36,45,50,55,58,62,68]	7 (28)	Delayed or frozen audio or video signal Technological glitches or system malfunction	“Several expressed frustration with the intermittent loss of connectivity and technical problems related to the videoconferencing during sessions.” [68]
	Protection of privacy and confidentiality [38,55,58,62,67]	5 (20)	Unclear privacy protection procedures Inability to confirm the legitimacy of the health care provider Inconsistent client health and personal information acts across different jurisdictions	“Ensuring participants are communicating with legitimate personnel, ensuring that the delivery format does not invade privacy.” [55]
	Limited clinical observations and communication [45,55,62]	3 (12)	Limited environmental information beyond the scope of the camera	“The therapist could not see the complete context in which the activity was performed...this made it difficult at times for the therapist to analyze performance breakdowns in order to guide the participants most effectively.” [55]

## Discussion

### Principal Findings

#### Overview

By summarizing what we know about structured, one-to-one videoconference intervention programs for people with chronic conditions, lessons can be gleaned for future development, execution, and feasibility. First, the findings of this review confirm what others have found: videoconference programs are both feasible and acceptable, often providing positive therapeutic benefits. Second, program purpose and objective may provide program developers and interventionists with important insights into program content and behavior change elements. Finally, while technology does overcome some barriers to access, it creates others.

#### Feasibility and Acceptability

Consistent with previous findings [69,70], this review found videoconference programs to be feasible and acceptable to clients, generating confidence for future program development. As reported by others [71,72], this review also found that spatial and temporal barriers, such as the need to travel to a hospital or clinic, were reduced or removed, and access to care for underserviced populations or areas was increased. Further evidence of participant acceptance was found in the high attendance and low attrition rates. In 67% (4/6) of the studies reporting completion rates, there was 100% completion, suggesting strong client uptake.

Reports on development of therapist-client rapport during videoconference sessions are mixed, with some indicating that providers struggle to build therapeutic alliances [26,73], whereas others indicate that videoconferencing does not hinder the therapist-client rapport or therapeutic alliance [73,74]. The studies in this review found that eye contact, facial expression, voice or tone, and other nonverbal communication were conveyed through the screen with high client satisfaction.

However, feasibility and acceptability were primarily based on qualitative data, client or health care provider impressions, or process evaluations. Future research to quantitatively assess the reduction in spatial and temporal barriers and the fidelity of therapist-client rapport are needed to confirm these findings.

#### Program Purpose Suggests Content and Behavior Change Elements

Analysis of program content (TEDSS domains) and behavior change strategies (BCT Taxonomy v1 groups) provides direction for health care providers and researchers developing or transferring in-person programs to internet-based format.

A total of 4 TEDSS domains and 4 BCT Taxonomy v1 groups dominated the 25 intervention programs. Over 50% of the programs (18/25, 72%; *disease and control* domain: 14/25, 56%; *healthy behavior* domain: 13/25, 52%; *internal* domain: 13/25, 52%) had content aligned with the same 4 domains (*disease control, healthy behaviors, internal, and process*) of the TEDSS framework, findings that mimic those of others using the TEDSS framework to analyze program content and program outcome measures [11,75]. However, potentially more instructive are the domains that received limited attention. Enhancing clients’ role management (ie, *activities* and *social interaction* domains) and strategies to find, access, or manage formal and informal supports and resources (ie, *resources* domain) are also part of managing and living with chronic conditions [76-78]. These content areas are also the very topics that clients identify as most important and least well covered [79]. Qualitative work by Satink et al [80], Audulv et al [81], Audulv [82], and others demonstrates how self-management needs change with time, particularly once clients return home and the focus on medical management diminishes. The paucity of intervention programs addressing clients’ role management has been highlighted in other reviews [11,75], as has the limited focus on finding and managing formal and informal resources [34]. On the basis of these findings, future development and adaptation of telehealth care programs could consider broadening the scope of program content and the alignment between client needs, particularly when clients are living in the community. Research to interrogate the relative importance of different content areas based on context, disease trajectory, and time since diagnosis would help identify active ingredients needed to tailor interventions.

BCT Taxonomy v1 techniques used in videoconferencing interventions appear to emulate asynchronous e-interventions for individuals with diabetes and programs for health promotion delivered using the internet or mobile phones [83-85]. Many techniques in the goals and planning and feedback and monitoring clusters, also found in this review, have had significant treatment effects in diabetes management [83-85]. Shaping knowledge and repetition and substitution (eg, practicing and rehearsing learned skills) also appeared to be common elements in the studies in this review, likely due to the inclusion criteria, which stressed active participation. Regardless, the results indicate that a wide array of behavior change techniques can be incorporated into telehealth care programs.

This review specifically excluded intervention programs based solely on repetition or intended for clients with mental health diagnoses. Despite this, the 2 most common program objectives were improving physical activity or function and improving mental health. Comparison of TEDSS domains and BCT Taxonomy v1 elements provides valuable insights for both in-person and telehealth care program development and delivery. While the analysis was descriptive and limited to these 2 program objectives (7/33, 21% of the studies each), marked differences were found, suggesting that program objectives have, at least up to now, driven content and behavior change strategies, 2 things often considered active ingredients in chronic disease management and intervention programs. These stark differences may reflect theoretical or disciplinary differences or perhaps the siloed delivery of health care. For example, differences in content related to the *healthy behaviors, internal, and social interaction* domains may at first seem intuitive. Greater emphasis on the *healthy behaviors* domain in programs focused on improving physical activity or function versus a focus on the *internal* and *social interaction* intuitively understandable. However, significant research links positive mental health outcomes to healthy eating, sleep hygiene, and exercise [86,87]. Similarly, improving physical activity or function in populations living with chronic conditions is often dependent on family and friends for support and motivation [88,89]. Similar contradictions seem to appear in the chosen BCT Taxonomy v1 strategies. Why feedback and monitoring strategies are more successful in building physical activity or function or why identity and regulation strategies are more important in programs that focus on mental health could be questioned. Research to disentangle these inconsistencies and questions is needed.

Regardless of the contradictions and questions, the finding that program content and techniques and, therefore, active ingredients vary by program objective has important implications for program development, training, and effectiveness studies. For example, the development of new intervention programs can be guided by knowledge of existing active ingredients. Moreover, using the TEDSS and BCT Taxonomy v1 in the planning stages may generate innovative programs. It is also important for future systematic reviews to avoid grouping programs with different objectives to avoid findings of average effectiveness across programs with different active ingredients.

Collectively, our findings demonstrate the value of using the well-defined TEDSS and BCT Taxonomy v1 frameworks to unify how interventions are reported and compared. Using common frameworks with defined terminology can potentially address the calls to identify and isolate content or active ingredients within complex interventions and assist providers in tailoring self-management interventions to client needs.

#### Challenges Delivering Interventions Using Videoconferencing

Despite the feasibility and acceptability of videoconference interventions, barriers related to technology literacy, compatible devices, and quality internet access were found. Evidence of barriers was 2-fold. First, many studies (18/33, 55%) excluded participants without a stable internet connection, compatible hardware, or family or social support at home. While it is unknown how many participants were excluded for these reasons, it is likely that they were marginalized populations or lived in rural areas where Wi-Fi connection is often less stable. This technological gap must be addressed to reduce health inequity using any telehealth care approach. Second, clients’ comfort level, confidence, and skills to effectively operate the necessary technology were the most common barriers reported despite efforts to provide initial training and the exclusion of participants without access. Careful planning, communication, and training are imperative to enhance comfort and confidence for service providers and clients [90]. The work by Vassilev et al [71] underscores this, finding “evidence that clients’ capability in technology use increased their propensity to benefit from interventions.” On the basis of the findings of this review, communicating with clients about confidence and preference of delivery format should be prioritized before the initial session.

Consistent with existing literature, technology breakdowns were a common cause of user frustration [91]. While 39% (13/33) of the studies verified the connectivity before intervention sessions, there were breakdowns or poor audio or video quality. Appointments had to be canceled or rescheduled through email or telephone. Although causes were unreported, limited bandwidth, software malfunction, and old add-on devices (eg, microphone or camera) are potential explanations. These findings recommend intentional preparation and contingency planning, such as a participant manual for troubleshooting common technology issues. Some highly qualified health care providers may also lack the technical skills or confidence to manage videoconference delivery. Adequate provider technology expertise should be assessed before service delivery via telehealth care formats [92]. Adding a technology support team member could meet a portion of this need and potentially enhance the therapeutic experience [93].

### Limitations and Future Research

Although this review covered a 20-year span up to January 2023, many interventions were planned or executed before the COVID-19 pandemic and may not capture the rapid development of videoconferencing services and policies after 2020. Technology for telehealth care and videoconferencing (eg, Zoom and Microsoft Teams) has dramatically advanced with the addition of products, video technology, and health care security (HIPAA compliance). Familiarity and comfort with technologies has also increased dramatically. Upcoming articles may reveal additional recommendations. The lack of information reported on security and confidentiality issues during videoconferencing suggests the need for a dedicated and in-depth study across countries and jurisdictions.

The findings of this review are limited to information reported in manuscripts and publicly available to researchers and providers. Authors were not contacted to clarify or confirm the content or active ingredients in the interventions. Therefore, reporting of content and behavior change strategies may be incomplete. More structured and uniform reporting is needed to overcome this gap. While scoping review methodology does not include the assessment of research quality or outcome evaluation, uniformity of reporting would benefit future systematic reviews or meta-analyses, helping identify links between active ingredients and outcomes. Finally, health care providers’ perspectives on videoconferencing could not be reported due to the small number of papers or studies reporting these data. The inherent provider-client collaboration in these interventions [10] warrants the need to also understand the challenges experienced by health care providers.

### Conclusions and Clinical Implications

Using videoconferencing to deliver one-to-one synchronous interventions is feasible and acceptable to clients. Health care providers can build positive therapeutic relationships via videoconference and simultaneously reduce temporal and physical barriers associated with access to care and improving reach to underserviced populations and areas. Despite this, barriers and concerns exist. Client confidence and comfort using technology, limited access to compatible and reliable hardware and internet connection, and technology breakdowns are common, highlighting the importance of technical support for providers and clients.

Structured, one-to-one videoconference interventions that actively engage people in the management of their chronic conditions remain primarily focused on medical and, to some extent, emotional management. Focused content to support integration into roles and everyday life, prioritize positive social interactions, and improve access to resources constitutes areas important to clients to strengthen interventions. The value of this content regardless of the program goal should be considered when developing future interventions. Similarly, additional behavior change techniques have potential to increase the effectiveness of interventions. With telehealth care practices expanding, further research is needed to point the way to best practice.

Moving in-person self-management interventions to internet-based formats must be intentional. While technology can reduce barriers, access to care based on device ownership and internet connectivity can be a delivery challenge. Health care teams may need to be expanded to include technical and technology support for providers and clients. Ensuring compliance with local privacy laws and protection of personal health information is required. Prepared contingency plans and a troubleshooting guide are strongly recommended in case of technology breakdowns. Existing program objectives appear to drive content and behavior change strategies, potentially limiting value to clients. Future programs could consider greater emphasis on enabling clients to find and organize formal and informal supports or manage their social interaction and daily activities, which are self-management domains that clients identify as important and poorly covered.

## Appendix

Multimedia Appendix 1 PRISMA-ScR (Preferred Reporting Items for Systematic Reviews and Meta-Analyses extension for Scoping Reviews) checklist.

Multimedia Appendix 2 Search strategies and number of articles found in each database.

Multimedia Appendix 3 Reviewer qualifications and involvement in the review process.

Multimedia Appendix 4 Videoconferencing hardware and software used in intervention programs for people with chronic conditions.

Multimedia Appendix 5 Intervention program content by Taxonomy of Every Day Self-Management Strategies and Behavior Change Technique Taxonomy version 1 categories.

## Abbreviations

BCT Taxonomy v1: Behavior Change Technique Taxonomy version 1
HIPAA: Health Insurance Portability and Accountability Act
PIPEDA: Personal Information Protection and Electronic Documents Act
PRISMA-ScR: Preferred Reporting Items for Systematic Reviews and Meta-Analyses extension for Scoping Reviews
TEDSS: Taxonomy of Every Day Self-Management Strategies

## Data Availability

The datasets generated during and analyzed during this study are not publicly available due to the complexity of manual coding and the need for related training but are available from the corresponding author on reasonable request.
