# Supplementary material for: Features of Structured, One-to-One Videoconference Interventions That Actively Engage People in the Management of Their Chronic Conditions: Scoping Review
Source: J Med Internet Res. 2025 Feb 26;27:e58543. doi: 10.2196/58543 (PMC11904366; doi:10.2196/58543)
Supplement: Multimedia Appendix 2 [file jmir_v27i1e58543_app2.pdf]

Search strategies and number of articles found in each database.

**Database and keywords**	**Source types**	**Publication date**	**Language**	**Age (population)**	**Number of articles found**
CINAHL					
Round 1<br>1. ((MM"Videoconferencing+") OR (MM "Teleconferencing")) OR AB ((videoconferenc* OR teleconferenc* OR virtual care OR skype)) OR TX ((videoconferenc* OR teleconferenc* OR virtual care OR skype))	Academic journals	Round 1: 2003-2020	English	All adult	Round 1: 1790
		Round 2: Jan 2020-Feb 2023			Round 2: 228
Medline					
1. ("Videoconferencing"[Mesh]) OR Telerehabilitation"[Mesh] OR Skype	Journal article	Round 1: 2003-2020	English	Adult 19+ years	Round 1: 929
		Round 2: 2020-2023			Round 2: 410
Embase					
1. videoconferencing/exp	Journal article	Round 1: 2003-2020	English	Adult	Round 1: 819
		Round 2: 2020-2023			Round 2: 928
PsycInfo					
1. (MM "Videoconferencing") OR (MM "Teleconferencing" OR MM "Videoconferencing" OR MM "Teleconsultation")<br>2. (health OR illness OR disease OR chronic condition OR patient)<br>1 & 2	Academic journals	Round 1: 2003-2020	English	All adult	Round 1: 229
		Round 2: June 2020-Feb 2023			Round 2: 88
OTSeeker					
1. [Title/Abstract] like 'videoconferenc* OR skype OR virtual care OR video call OR telehealth OR telerehab* OR ehealth OR digital'		(Cannot specify dates; articles are screened manually; Articles after 2020 manually searched)			Round 1: 48
					Round 2: 0
PEDro					

1. videoconferenc*					Round 1: 22
					Round 2: 13
Cochrane					
1. Trials matching MeSH descriptor: [Videoconferencing] explode all trees					Round 1: 197
					Round 2: 52
ERIC					
1. (videoconferenc* OR teleconferenc* OR skype) AND (health OR illness OR disease OR chronic condition OR patient)<br>2. (health OR illness OR disease OR chronic condition OR patient)<br>1 & 2	Peer reviewed scholarly journals	Round 1: 2003-2020	English	Adult education OR post secondary education OR higher education OR High School Equivalency Programs	Round 1: 33
		Round 2: June 2020-Feb 2023			Round 2: 5
