# Supplementary material for: Features of Structured, One-to-One Videoconference Interventions That Actively Engage People in the Management of Their Chronic Conditions: Scoping Review
Source: J Med Internet Res. 2025 Feb 26;27:e58543. doi: 10.2196/58543 (PMC11904366; doi:10.2196/58543)
Supplement: Multimedia Appendix 3 [file jmir_v27i1e58543_app3.pdf]

Reviewer qualifications and involvement in the review process.

Reviewer	Reviewer qualifications	Involvement in review
1	Professional master’s	Initial search
2	PhD student	Initial search
3	Post doctoral fellow	Initial and updated searches
4	Professional doctorate	Initial and updated searches
5	PhD student	Initial and updated searches
6	Senior researcher	Initial and updated searches
7	Professional master’s	Updated search
8	Senior researcher	Updated search
