# Supplementary material for: Features of Structured, One-to-One Videoconference Interventions That Actively Engage People in the Management of Their Chronic Conditions: Scoping Review
Source: J Med Internet Res. 2025 Feb 26;27:e58543. doi: 10.2196/58543 (PMC11904366; doi:10.2196/58543)
Supplement: Multimedia Appendix 4 [file jmir_v27i1e58543_app4.pdf]

Videoconferencing hardware and software used in intervention programs for people with chronic conditions.

Authors (year)	Hardware		Software
	Device	Source	App or platform
Alencar et al. (2019)	Apple iPhone	NR	Amwell®
Alencar et al. (2020)	Apple iPhone	NR	Amwell®
Aubin et al. (2019)	NR	NR	Skype™
Brunet et al. (2022)	NR	NR	Zoom®
Chemtob et al. (2019)	Computer	Participants’ possession	REACTS®
Cruice et al. (2021)	iPad/Tablet OR Computer	Provided by researcher if participants did not have a device	Skype™
Garland et al. (2021)	Available and suitable device	NR	Zoom®
Gilboa et al. (2019)	iPad/Tablet OR Computer	Participants’ possession	Skype™
Goren et al. (2022)	NR	NR	NR
Hastings et al. (2021)	iPad/Tablet	Provided by researcher if participants did not have a device	VA Video Connect (VVC)
Kelleher et al. (2019)	iPad/Tablet	Researchers provided	NR
Kline et al. (2019)	iPad/Tablet	Researchers provided	NR
Lafaro et al. (2020)	NR	NR	Zoom®
Lavelle et al. (2022)	NR	NR	Zoom®
Lawson et al. (2020)	NR	NR	Zoom®
Lawson et al. (2022)	iPad/Tablet OR Computer	NR	Zoom®
Lynch et al. (2016)	iPad/Tablet	Researchers provided	Web-based portal
Milbury et al. (2020)	iPad/Tablet	Researchers provided OR Participants’ possession depending on preference	FaceTime
Miller et al. (2017)	iPad/Tablet	Researchers provided	NR
Ng et al. (2013)	NR	Researchers provided	Skype™ Professional
Ownsworth et al. (2019)	Available and suitable device	Participants’ possession	Zoom®
Pfammatter et al. (2022)	iPhone/ Smartphone	Participants’ possession	Intervention Specific App
Rietdijk et al. (2019)	NR	NR	Skype™
Rietdijk et al. (2020)	Computer	Participants’ possession	Skype™
Somers et al. (2015)	iPad/Tablet	Researchers provided	Skype™
Tanenbaum et al. (2021)	NR	NR	Zoom®

Vellani et al. (2022)	Available and suitable device	Researchers provided	Microsoft Teams
Winger et al. (2020)	NR	NR	NR
Winger et al. (2022)	iPad/Tablet	Researchers provided	Skype™
Wood et al. (2021)	iPad/Tablet	Researchers provided	FaceTime
Ymer et al. (2021)	NR	NR	NR
Yosef et al. (2022)	NR	NR	Zoom® OR Skype™
Yuen (2013)	Computer	Provided by researcher if participants did not have a device	SCOPIA®
