# Supplementary material for: Features of Structured, One-to-One Videoconference Interventions That Actively Engage People in the Management of Their Chronic Conditions: Scoping Review
Source: J Med Internet Res. 2025 Feb 26;27:e58543. doi: 10.2196/58543 (PMC11904366; doi:10.2196/58543)
Supplement: Multimedia Appendix 5 [file jmir_v27i1e58543_app5.pdf]

Intervention program content by Taxonomy of Every Day Self-Management Strategies and Behavior Change Technique Taxonomy version 1 categories.

Intervention	BCT	TEDSS
physicAl aCtivity Counselling for young adult cancEr SurvivorS (ACCESS)	Goals and planning<br>Feedback and monitoring<br>Social support<br>Shaping knowledge<br>Regulation<br>Natural consequences<br>Antecedents	Healthy behaviours<br>Process<br>Internal<br>Activities
Online supported conversation (SC) for participation intervention	Goals and planning<br>Social support<br>Shaping knowledge<br>Repetition and substitution<br>Antecedents	Process<br>Social interactions<br>Resource
Cognitive Behaviour Therapy for Insomnia (CBT-I)	Feedback and monitoring<br>Regulation<br>Associations<br>Identity<br>Antecedents	Healthy Behaviours<br>Internal<br>Process
COgnitive Behavioral and MINdfulness-based stress reduction with Daily EXercise (COBMINDEX)	Shaping knowledge<br>Regulation<br>Natural consequences<br>Repetition and substitution<br>Identity<br>Antecedents	Internal
Telehealth Perioperative Physical Activity Intervention	Goals and planning<br>Feedback and monitoring<br>Social support<br>Natural consequences<br>Antecedents	Healthy behaviours<br>Process
Brief and telehealth Acceptance and Commitment Therapy (ACT)	Goals and planning<br>Shaping knowledge<br>Regulation<br>Associations<br>Natural consequences	Internal<br>Disease control<br>Process

	Repetition and substitution	
	Identity	
Couple-Based Meditation (CBM)	Shaping knowledge	Disease control
	Regulation	Social interactions
	Identity	Internal
	Self-belief	Resource
Elements Vital to treat Obesity (EVO)	Goals and planning	Healthy behaviours
	Feedback and monitoring	Process
	Shaping knowledge	
ONBOARD (Overcoming Barriers and Obstacles to Adopting Diabetes Devices)	Goals and planning	Disease control
	Shaping knowledge	Process
	Comparison of behaviours	
	Self-belief	
Voice Your Values (VYV)	Shaping knowledge	Resource
	Identity	Social interactions
		Internal
Tele-Nutrition Counseling Program	Goals and planning	Healthy behaviours
	Feedback and monitoring	Process
	Shaping knowledge	
	Repetition and substitution	
	Self-belief	
Cognitive Behavioral Therapy for Sleep Disturbance and Fatigue (CBT-SF)	Feedback and monitoring	Healthy Behaviours
	Regulation	Disease Control
	Associations	Process
	Identity	Internal
	Antecedents	Activities
Care Management Program	Antecedents	Healthy behaviours
		Disease control
Psychosocial intervention	Goals and planning	Internal
	Feedback and monitoring	Social interactions
	Shaping knowledge	Disease control
	Regulation	
	Repetition and substitution	
	Identity	
Leisure Time Physical Activity (LTPA)	Goals and planning	Process
	Feedback and monitoring	Healthy behaviours
	Social support	

	Comparison of behaviours<br>Comparison of outcomes<br>Identity<br>Self-belief	
Behavior change intervention	Goals and planning<br>Feedback and monitoring<br>Social support<br>Shaping knowledge<br>Repetition and substitution	Process<br>Healthy behaviours
Tablet-Aided BehavioraL intervention EffecT on Self-management skills (TABLETS)	Goals and planning<br>Feedback and monitoring<br>Shaping knowledge<br>Repetition and substitution	Resource<br>Process<br>Internal<br>Healthy behaviours<br>Disease control
Physical Activity Behaviour Change (PABC)	Goals and planning<br>Feedback and monitoring<br>Social support<br>Shaping knowledge<br>Repetition and substitution	Process<br>Healthy behaviours<br>Disease control
Making Sense of Brain Tumour program (Tele-MAST)	Goals and planning<br>Shaping knowledge<br>Regulation	Process<br>Internal<br>Social interactions<br>Disease control
Telecare on oral health	Goals and planning<br>Feedback and monitoring<br>Social support<br>Shaping knowledge<br>Comparison of behaviours<br>Repetition and substitution<br>Antecedents<br>Self-belief	Process<br>Activities<br>Disease control
Telehealth-Enabled Approach to Multidisciplinary Care (TEAM)	Goals and planning<br>Feedback and monitoring<br>Shaping knowledge<br>Associations<br>Repetition and substitution	Process<br>Internal<br>Healthy behaviours<br>Disease control
	Goals and planning	Process

Cognitive Orientation to daily Occupational Performance (CO-OP)	Feedback and monitoring<br>Repetition and substitution	Activities
Pain Coping Skill Training (PCST)	Goals and planning<br>Shaping knowledge<br>Regulation<br>Repetition and substitution<br>Identity<br>Self-belief<br>Feedback and monitoring<br>Social support<br>Natural consequences<br>Comparison of behaviours	Internal<br>Process<br>Activities<br>Disease control
Modified Monash memory skills program	Shaping knowledge<br>Repetition and substitution<br>Goals and planning<br>Goals and planning	Internal<br>Healthy behaviours<br>Disease control
TBIconneCT	Antecedents<br>Goals and planning<br>Feedback and monitoring<br>Shaping knowledge<br>Comparison of behaviours<br>Repetition and substitution<br>Comparison of outcomes	 Social interactions<br>Disease control
